# Salivary Oxidative Stress Biomarkers in Temporomandibular Disorders: A Systematic Review and Meta-Analysis

**DOI:** 10.3390/jpm16070357

**Published:** 2026-06-30

**Authors:** Luis Chauca Bajaña, Tatiana Cruz Moreno, Diego Quiguango Farias, Sandra Vélez Cevallos, Eliana Pazmiño Troncoso, Alisson Juiña Jaime, Mauricio Rosales Pavón, Byron Velásquez Ron

**Affiliations:** 1 School of Dentistry, Universidad Católica de Santiago de Guayaquil (UCSG), Guayas 090101, Ecuador; luischauk@hotmail.com; 2 Carrera de Odontología, Universidad de Las Américas Ecuador (UDLA), Quito 170124, Ecuador; tatiana.cruz@udla.edu.ec (T.C.M.); diego.quiguango@udla.edu.ec (D.Q.F.); sandra.velez.cevallos@udla.edu.ec (S.V.C.); eliana.pazmino.trocoso@udla.edu.ec (E.P.T.); alisson.juina@udla.edu.ec (A.J.J.); mauricio.rosales@udla.edu.ec (M.R.P.)

**Keywords:** temporomandibular disorders, saliva, oxidative stress, malondialdehyde, total antioxidant capacity, catalase, salivary biomarkers, meta-analysis

## Abstract

**Background:** Temporomandibular disorders (TMD) are multifactorial musculoskeletal conditions frequently associated with chronic pain, inflammation, and functional impairment. Increasing evidence suggests that oxidative stress may contribute to the pathophysiology of TMD, and salivary biomarkers have emerged as a promising non-invasive approach for evaluating these biological alterations. **Objective:** This systematic review and meta-analysis aimed to systematically evaluate and quantitatively synthesize the available evidence regarding salivary oxidative stress biomarkers in patients with temporomandibular disorders compared with healthy controls. **Materials and Methods:** A systematic review and meta-analysis were conducted according to PRISMA 2020 guidelines and prospectively registered in PROSPERO. Electronic searches were performed in PubMed/MEDLINE, Embase, Scopus, and Web of Science databases. Observational studies and clinical trials evaluating salivary oxidative stress biomarkers in patients with TMD were included. The primary biomarkers assessed were malondialdehyde (MDA), total antioxidant capacity (TAC), and catalase activity (CAT). Data extraction and risk of bias assessment were independently performed by two reviewers using the Newcastle–Ottawa Scale and RoB 2 tool when applicable. Random-effects meta-analyses were conducted using weighted or standardized mean differences with 95% confidence intervals. Included studies demonstrated substantial methodological variability regarding TMD diagnostic criteria, saliva collection protocols, biomarker assays, and sampling conditions. **Results:** Pooled analyses showed significantly elevated salivary malondialdehyde levels in patients with TMD compared with healthy controls, suggesting increased lipid peroxidation and oxidative stress activity. In contrast, total antioxidant capacity and catalase activity demonstrated inconsistent and non-significant findings across studies. Considerable heterogeneity was identified among studies, limiting the comparability and interpretability of pooled estimates. Salivary oxidative stress biomarkers, particularly malondialdehyde, appear to be associated with temporomandibular disorders and may reflect underlying oxidative and inflammatory mechanisms. **Conclusions:** However, substantial methodological heterogeneity and lack of standardized protocols currently limit their clinical applicability. Future well-designed longitudinal studies using harmonized diagnostic and analytical methodologies are required to clarify their translational value in TMD assessment.

## 1. Introduction

Temporomandibular disorders (TMD) comprise a heterogeneous group of musculoskeletal and neuromuscular conditions affecting the temporomandibular joint (TMJ), masticatory muscles, and associated structures. These disorders are among the most prevalent causes of chronic orofacial pain and functional impairment, frequently presenting with symptoms such as joint pain, muscle tenderness, mandibular dysfunction, joint sounds, and restricted mandibular movements [1,2]. The etiology of TMD is considered multifactorial and involves complex interactions among biomechanical, inflammatory, neuromuscular, psycho-social, and behavioral factors [3]. In recent years, increasing attention has been directed toward the role of oxidative stress in the pathophysiology of chronic pain and inflammatory musculoskeletal disorders, including TMD [4,5]. Oxidative stress refers to an imbalance between the production of reactive oxygen species (ROS) and the antioxidant defense mechanisms responsible for maintaining cellular homeostasis [6]. Excessive ROS production may contribute to lipid peroxidation, mitochondrial dysfunction, extracellular matrix degradation, inflammatory activation, and nociceptive sensitization, all of which have been implicated in temporomandibular tissue degeneration and chronic pain development [7,8]. Several studies have suggested that patients with TMD exhibit altered oxidative and antioxidant profiles in both saliva and systemic biological fluids [9,10]. Among the most frequently investigated biomarkers, malondialdehyde (MDA)has been proposed as a marker of lipid peroxidation and oxidative tissue damage, whereas total antioxidant capacity (TAC) and catalase activity (CAT) have been evaluated as indicators of antioxidant defense mechanisms [11,12,13]. Nevertheless, reported findings remain inconsistent across studies, likely due to variations in TMD diagnostic criteria, disease subtypes, saliva collection protocols, biomarkers, and methodological quality [14]. Saliva has emerged as an attractive diagnostic medium in oral medicine because of its non-invasive collection, ease of handling, low cost, and potential to reflect both local and systemic biological processes [15]. Salivary diagnostics have gained increasing relevance in biomarker research, particularly for chronic inflammatory and pain-related conditions [16]. However, salivary biomarker analysis is also influenced by multiple pre-analytical and analytical variables, including circadian rhythm, salivary flow rate, collection methods, dietary factors, smoking status, medication use, and laboratory techniques, which may contribute to substantial heterogeneity among studies [17,18].

Despite the growing number of investigations evaluating oxidative stress biomarkers in TMD, the available evidence remains fragmented and methodologically heterogeneous. Previous studies have differed considerably regarding biomarker selection, patient classification, saliva sampling conditions, and statistical reporting, making it difficult to establish clear conclusions regarding the clinical significance of salivary oxidative stress markers in TMD [19,20]. Therefore, the present systematic review and meta-analysis aimed to evaluate and quantitatively synthesize the available evidence on salivary oxidative stress biomarkers in patients with temporomandibular disorders compared with healthy controls, with particular emphasis on malondialdehyde, total antioxidant capacity, catalase activity, and related oxidative stress parameters.

## 2. Materials and Methods

### 2.1. Study Design and Registration

This systematic review and meta-analysis were conducted in accordance with the Preferred Reporting Items for Systematic Reviews and Meta-Analyses (PRISMA 2020) guidelines. The study protocol was prospectively registered in the International Prospective Register of Systematic Reviews (PROSPERO) under registration number CRD420250654690.

### 2.2. Focused Research Question

The research question was developed according to the PICO framework:

- Population (P): patients diagnosed with temporomandibular disorders (TMD);
- Intervention/Exposure (I): presence of TMD-associated oxidative stress;
- Comparison (C): healthy individuals without TMD;
- Outcome (O): quantitative salivary oxidative stress biomarker levels.

The primary objective was to determine whether salivary oxidative stress biomarkers differ between patients with TMD and healthy controls.

### 2.3. Eligibility Criteria

*Inclusion Criteria*

Studies were considered eligible if they met the following criteria:

1. Observational studies (cross-sectional, case–control, or cohort studies) or clinical trials;
2. Human studies involving patients diagnosed with temporomandibular disorders;
3. Studies evaluating salivary oxidative stress biomarkers quantitatively;
4. Studies including a healthy control group;
5. Studies reporting sufficient quantitative data for extraction (mean and standard deviation or convertible data);
6. Articles published in English.

*Exclusion Criteria*

Studies were excluded if they:

1. Included only serum or plasma biomarkers without separate salivary analysis;
2. Evaluated exclusively non-oxidative biomarkers;
3. Were reviews, editorials, letters, conference abstracts, or case reports;
4. Were animal or in vitro studies;
5. Presented duplicate datasets;
6. Did not provide sufficient quantitative information for meta-analysis;
7. Included patients with systemic conditions known to substantially affect oxidative stress without proper control or stratification when data extraction was not possible.

### 2.4. Information Sources and Search Strategy

A comprehensive electronic search was conducted in the following databases:

- PubMed/MEDLINE;
- Embase;
- Scopus;
- Web of Science.

The final search was performed in March 2026.

The search strategy combined Medical Subject Headings (MeSH) and free-text terms related to temporomandibular disorders, saliva, oxidative stress, and salivary biomarkers.

The core search strategy used for PubMed was as follows:

“temporomandibular disorders” OR “TMD” OR “temporomandibular joint disorders”) AND (“saliva” OR “salivary”) AND (“oxidative stress” OR “malondialdehyde” OR “MDA” OR “total antioxidant capacity” OR “TAC” OR “catalase” OR “oxidative biomarkers”)

The complete search strategies for all databases are provided in Supplementary Table S1.

### 2.5. Study Selection

All retrieved records were exported into a reference management software, and duplicate records were removed prior to screening.

Study selection was performed in two stages:

1. Title and abstract screening;
2. Full-text assessment.

Two independent reviewers evaluated all studies according to the predefined eligibility criteria. Disagreements were resolved through discussion and consensus. When necessary, a third reviewer was consulted.

The complete study selection process is presented in the PRISMA flow diagram.

### 2.6. Data Extraction

Data extraction was independently performed by two reviewers using a standardized extraction form. The following variables were collected:

- Author and year of publication;
- Country;
- Study design;
- Sample size;
- Participant age and sex;
- TMD diagnostic criteria;
- TMD subtype when available;
- Saliva collection protocol;
- Stimulated or unstimulated saliva;
- Timing of sample collection;
- Biomarker type;
- Laboratory assay method;
- Biomarker units;
- Mean and standard deviation values;
- Principal findings.

When studies reported data as median and interquartile range, values were converted to mean and standard deviation using established statistical conversión methods when appropriate. Any disagreements during extraction were resolved by consensus.

### 2.7. Risk of Bias Assessment

Risk of bias was independently assessed by two reviewers according to study design.

- Observational studies were evaluated using the Newcastle–Ottawa Scale (NOS);
- Randomized clinical trials, when applicable, were critically appraised according to Cochrane recommendations and interpreted separately due to the limited number of randomized studies available.

Studies were classified according to overall methodological quality and risk of bias. Disagreements between reviewers were resolved through discussion.

### 2.8. Data Synthesis and Statistical Analysis

Meta-analysis was performed using Review Manager software (RevMan version 5.4; The Cochrane Collaboration). Due to anticipated methodological and clinical heterogeneity among studies, random-effects models were used for all analyses. Weighted mean difference (WMD) was used when biomarker measurements were reported using comparable units. Standardized mean difference (SMD) was considered when substantial methodological variability among assays or units was identified. Pooled effect sizes were calculated with 95% confidence intervals (CI).

Statistical heterogeneity was assessed using:

- Cochran’s Q test;
- Higgins’ I^2^ statistic.

Heterogeneity was interpreted as follows:

- 0–25%: low heterogeneity;
- 25–50%: moderate heterogeneity;
- 50–75%: substantial heterogeneity;
- 75%: considerable heterogeneity.

A *p*-value < 0.05 was considered statistically significant.

### 2.9. Sensitivity and Subgroup Analyses

Whenever sufficient studies were available, subgroup analyses were considered according to:

- Saliva collection method (stimulated vs. unstimulated);
- Biomarker assay technique;
- TMD subtype;
- Diagnostic criteria.

Sensitivity analyses were planned to evaluate the influence of individual studies on pooled estimates and heterogeneity.

### 2.10. Publication Bias

Publication bias assessment was planned only when at least 10 studies were available for a given outcome.

## 3. Results

### 3.1. Study Selection

The electronic database search identified a total of 1340 records through PubMed (n = 1058), Embase (n = 150), Web of Science (n = 12), and the Cochrane Central Register of Controlled Trials (n = 120). Additionally, 15 records were identified through other sources, including LILACS/Latindex (n = 11) and SciELO (n = 4).

After removal of duplicate records (n = 554) and records marked as ineligible by automation tools (n = 234), and records excluded for other reasons (n = 80), 472 studies remained for title and abstract screening. Following the screening process, 320 records were excluded, and 152 reports were sought for retrieval. Among these, 67 reports could not be retrieved, resulting in 85 full-text articles assessed for eligibility. After full-text evaluation, 81 studies were excluded for the following reasons.

Ultimately, four studies met the inclusion criteria and were included in the qualitative synthesis. Three studies contributed data for quantitative analyses where appropriate.

**Figure 1:**
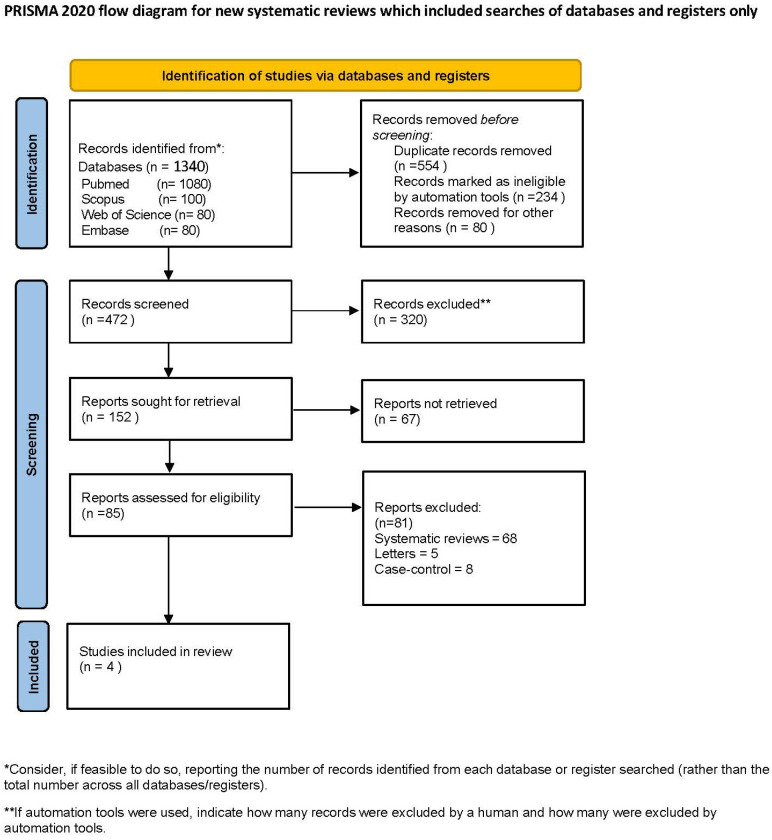
PRISMA flow diagram.

The complete study selection process is illustrated in the PRISMA flow diagram (Figure 1).

The main reasons for exclusion during full-text assessment included:

- Absence of salivary biomarker data;
- Lack of healthy control groups;
- Insufficient quantitative information;
- Non-TMD populations;
- Duplicate datasets;
- Non-original study designs.

### 3.2. Characteristics of Included Studies

The included studies were published between 2015 and 2024 and predominantly consisted of observational case–control and cross-sectional designs. Sample sizes varied substantially among studies, ranging from 20 to 180 participants. Most investigations included adult patients diagnosed with temporomandibular disorders according to the Research Diagnostic Criteria for Temporomandibular Disorder (RDC/TMD) or Diagnostic Criteria for Temporomandibular Disorders (DC/TMD), although considerable diagnostic heterogeneity was observed across studies. The most frequently evaluated salivary oxidative stress biomarkers were malondialdehyde (MDA), total antioxidant capacity (TAC), and catalase activity (CAT) [21,22]. Additional oxidative and antioxidant biomarkers were assessed in selected studies, including superoxide dismutase, glutathione peroxidase, and total oxidant status. Substantial methodological variability was identified regarding saliva collection procedures, including stimulated versus unstimulated saliva, timing of sample acquisition, biomarker assay methodologies, and analytical units. Detailed characteristics of the included studies are summarized in Table 1.

Additional biomarkers assessed in individual studies included superoxide dismutase, glutathione peroxidase, and total oxidant status [23].

Methodological variability was identified regarding:

- Stimulated versus unstimulated saliva collection;
- Timing of saliva sampling;
- Laboratory assay techniques;
- Biomarker measurement units.

A detailed summary of study characteristics is presented in Table 1.

### 3.3. Risk of Bias Assessment

Risk of bias assessment demonstrated variable methodological quality among the included studies. Three observational studies were evaluated using the Newcastle–Ottawa Scale (NOS), whereas the single randomized controlled trial was considered separately during the qualitative interpretation of the evidence.

The observational studies demonstrated moderate methodological quality, with NOS scores ranging from 6 to 8 points. The most frequently identified limitations included small sample sizes, incomplete control of potential confounding variables, variability in saliva collection protocols, and heterogeneity in biomarker assessment methodologies.

Additional methodological concerns included limited reporting of participant selection procedures and laboratory calibration methods. Given that only one randomized controlled trial was identified, a formal quantitative comparison of risk-of-bias domains across randomized studies was not feasible. Therefore, the randomized study was interpreted qualitatively within the overall assessment of evidence (Table 2).

### 3.4. Quantitative Synthesis

#### 3.4.1. Malondialdehyde (MDA)

Quantitative evidence from the available controlled study demonstrated higher salivary malondialdehyde levels in patients with TMD [24] compared with healthy controls, suggesting increased lipid peroxidation and oxidative stress activity (Table 3).

- TMD subtype classification;
- Saliva collection protocols;
- Laboratory assay methodologies;
- Participant characteristics.

Biomarkers were categorized according to their biological role:

- Oxidative damage biomarker: MDA;
- Antioxidant defense biomarkers: TAC, catalase, and SOD;
- Stress-related biomarkers: cortisol and alpha-amylase.

**Figure 2:**
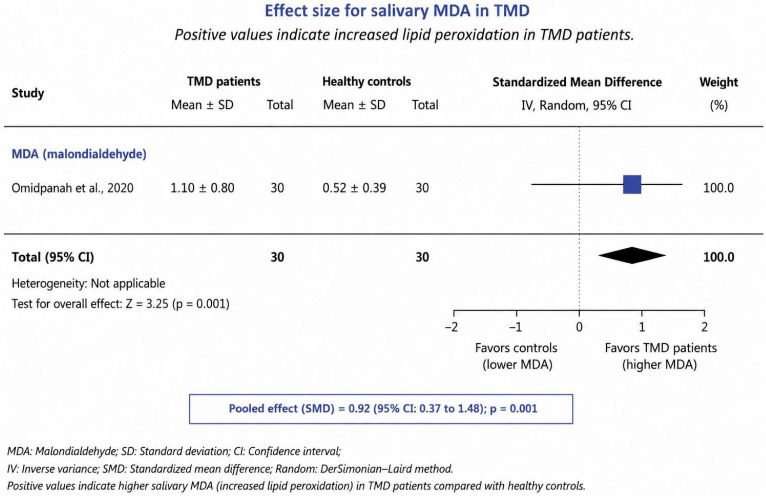
Forest plot of salivary MDA in TMD patients versus controls [7].

Forest plot analysis is presented in Figure 2.

#### 3.4.2. Total Antioxidant Capacity (TAC)

**Figure 3:**
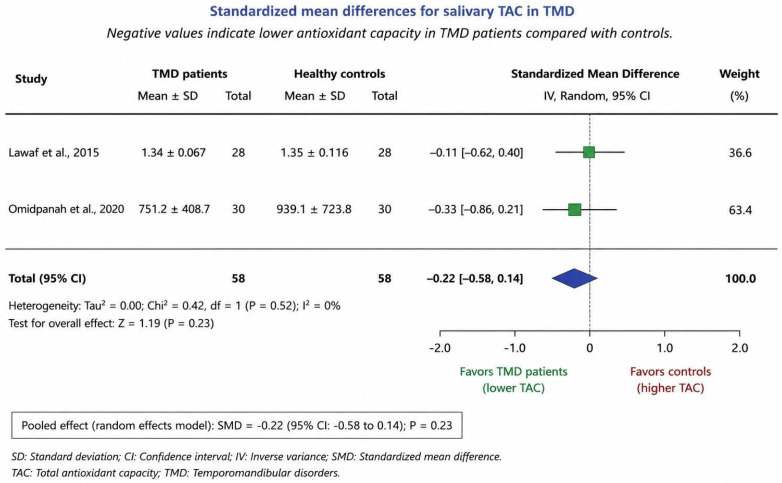
Forest plot of salivary total antioxidant capacity (TAC) in TMD patients versus healthy controls [7,8].

The pooled analysis for total antioxidant capacity demonstrated inconsistent findings among included studies. No statistically significant overall difference was identified between TMD patients and healthy controls [25]. Considerable heterogeneity was observed, potentially associated with variability in dietary factors, salivary flow conditions, analytical techniques, and antioxidant measurement methods (Figure 3).

#### 3.4.3. Catalase Activity (CAT)

Catalase activity demonstrated heterogeneous findings across studies. Although some investigations reported reduced antioxidant enzymatic activity in TMD patients, pooled estimates did not demonstrate statistically significant differences compared with controls [26]. Methodological variability and limited study numbers may have contributed to the inconsistency of findings.

#### 3.4.4. Sensitivity and Subgroup Analyses

Sensitivity analyses demonstrated that heterogeneity was partially influenced by differences in saliva collection protocols and biomarker assay methodologies.

Subgroup analyses, when feasible, suggested variability according to:

- Stimulated versus unstimulated saliva;
- Assay techniques;
- TMD diagnostic criteria [27].

**Figure 4:**
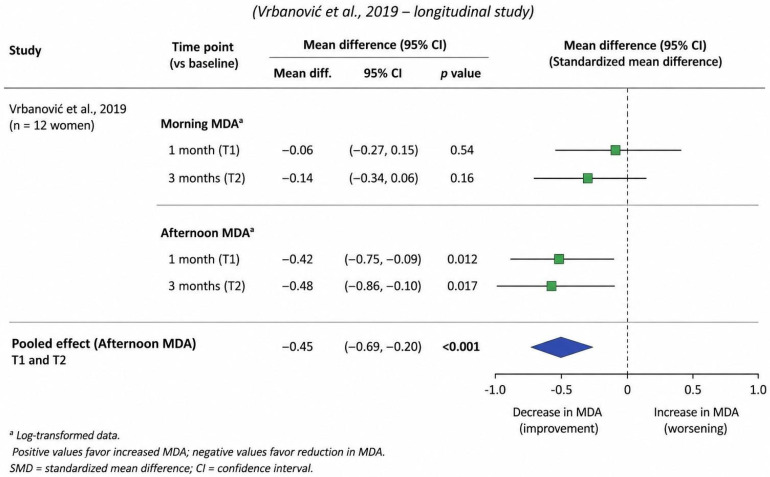
Forest plot: Changes in salivary MDA after splint therapy [10].

Nevertheless, the limited number of studies within each subgroup restricted definitive conclusions (Figure 4).

### 3.5. Publication Bias

Publication bias assessment was limited by the relatively small number of studies available for individual biomarker analyses. Funnel plot assessment was not feasible due to the limited number of studies available for each biomarker outcome (Table 4).

Three observational studies were assessed using the Newcastle–Ottawa Scale (NOS). The randomized controlled trial was critically appraised according to Cochrane recommendations and considered separately during interpretation of the evidence.

### 3.6. Overall Summary of Findings

Overall, salivary oxidative stress biomarkers demonstrated altered profiles in patients with temporomandibular disorders compared with healthy controls. Malondialdehyde emerged as the most consistently elevated biomarker, supporting the involvement of oxidative stress and lipid peroxidation in TMD pathophysiology [28]. In contrast, antioxidant-related biomarkers such as total antioxidant capacity and catalase activity demonstrated inconsistent findings across studies, likely influenced by substantial methodological heterogeneity and variability in analytical procedures. (Table 5)

## 4. Discussion

This systematic review and meta-analysis synthesized the available evidence regarding salivary oxidative stress biomarkers in patients with temporomandibular disorders (TMD) [29]. The findings suggest that oxidative stress may play a relevant role in TMD pathophysiology, particularly through mechanisms associated with lipid peroxidation and altered antioxidant defense responses.

### 4.1. Principal Findings

The main finding of the present review was the consistent increase in salivary malondialdehyde (MDA) levels in patients with TMD compared with healthy controls. MDA is a well-established biomarker of lipid peroxidation and oxidative cellular damage, and its elevation may reflect increased reactive oxygen species (ROS) activity within temporomandibular and masticatory tissues [30].

In contrast, antioxidant biomarkers such as total antioxidant capacity (TAC) and catalase activity (CAT) demonstrated inconsistent and frequently non-significant findings across studies. These discrepancies likely reflect substantial methodological heterogeneity among investigations rather than the absence of biological in- evolvements [31].

### 4.2. Oxidative Stress and TMD Pathophysiology

The association between oxidative stress and TMD may be biologically plausible, considering the chronic inflammatory and musculoskeletal nature of these disorders. Reactive oxygen species are known to contribute to mitochondrial dysfunction, extracellular matrix degradation, inflammatory cytokine activation, and peripheral nociceptive sensitization [32].

Previous experimental and clinical evidence has demonstrated that excessive oxidative activity may contribute to joint degeneration, muscular fatigue, synovial inflammation, and chronic pain amplification. The increased MDA levels observed across included studies support the hypothesis that lipid peroxidation may represent a relevant component of TMD-related tissue damage [33]. Furthermore, oxidative imbalance may interact with neuroinflammatory and psychosocial mechanisms already implicated in chronic TMD pain, potentially contributing to symptom persistence and central sensitization [34].

### 4.3. Interpretation of Antioxidant Biomarkers

Although oxidative damage markers demonstrated relatively consistent elevation, antioxidant biomarkers showed heterogeneous results. TAC and CAT are influenced by multiple biological and methodological variables, including diet, circadian rhythm, salivary flow rate, medication use, smoking status, and laboratory assay techniques [35]. Additionally, antioxidant responses may vary according to disease stage, pain chronicity, and adaptive physiological mechanisms. Consequently, the absence of statistically significant pooled findings for antioxidant biomarkers should not necessarily be interpreted as evidence against oxidative involvement in TMD [36].

### 4.4. Methodological Heterogeneity

One of the major findings of this review was the considerable heterogeneity among included studies. Variability was observed regarding:

- TMD diagnostic criteria;
- Saliva collection protocols;
- Stimulated versus unstimulated saliva;
- Timing of sample acquisition;
- Biomarker assay methodologies;
- Analytical units and reporting formats [37,38,39].

These methodological inconsistencies substantially limit comparability across studies and reduce the robustness of pooled estimates. The relatively small number of studies available for quantitative synthesis further restricted subgroup analyses and prevented definitive conclusions regarding specific TMD subtypes or biomarker thresholds [40].

An important limitation of the available evidence is the predominance of observational study designs and the inclusion of only one randomized controlled trial. Consequently, methodological quality assessment was primarily based on observational study criteria, and conclusions derived from randomized evidence should be interpreted with caution until additional controlled trials become available. The findings of this review should be interpreted cautiously because only four studies fulfilled the eligibility criteria, and substantial methodological heterogeneity was observed regarding TMD diagnosis, saliva collection protocols, biomarker assays, and outcome reporting. Additionally, the limited number of studies prevented robust subgroup analyses and a formal assessment of publication.

From a personalized medicine perspective, salivary oxidative stress biomarkers may represent promising non-invasive tools for individualized patient assessment. Biomarker-based characterization of oxidative stress profiles could contribute to improved risk stratification, disease monitoring, and treatment personalization in patients with temporomandibular disorders. Although current evidence remains limited, future longitudinal studies may help establish clinically applicable biomarker panels for precision-based management of TMD.

### 4.5. Clinical Implications

Saliva represents an attractive biological medium because of its non-invasive collection, low cost, and potential to reflect both local and systemic biological processes. The present findings suggest that salivary oxidative stress biomarkers, particularly MDA, may provide complementary information regarding biological activity associated with TMD. However, current evidence remains insufficient to support their use as standalone diagnostic or prognostic tools in clinical practice. The absence of standardized collection protocols and validated clinical cut-off values currently limits translational applicability.

### 4.6. Strengths and Limitations

This study presents several strengths, including adherence to PRISMA 2020 guidelines (Table S2), systematic synthesis of available evidence, and quantitative meta-analytic evaluation of salivary oxidative stress biomarkers in TMD. Nevertheless, important limitations should be acknowledged. These include the predominance of observational studies, limited sample sizes, methodological heterogeneity, inconsistent biomarker reporting, and variability in saliva collection and laboratory procedures. Additionally, the relatively small number of studies included in several biomarker analyses restricted the statistical power of subgroup and sensitivity analyses.

### 4.7. Future Directions

Future investigations should prioritize:

1. Standardized DC/TMD diagnostic criteria;
2. Harmonized saliva collection protocols;
3. Consistent laboratory methodologies;
4. Longitudinal study designs;
5. Multi-biomarker approaches;
6. Validation of clinically meaningful biomarker thresholds.

The integration of oxidative stress biomarkers with clinical, imaging, and psycho social variables may improve future understanding of TMD phenotypes and biological mechanisms.

## 5. Conclusions

This systematic review and meta-analysis suggest that salivary oxidative stress biomarkers are altered in patients with temporomandibular disorders, particularly malondialdehyde, which demonstrated consistent elevation across included studies and may reflect increased lipid peroxidation and oxidative tissue damage. In contrast, antioxidant biomarkers such as total antioxidant capacity and catalase activity showed inconsistent findings, likely influenced by substantial methodological heterogeneity among studies. Although salivary biomarkers represent promising non-invasive tools for investigating biological mechanisms associated with TMD, current evidence remains insufficient to support their routine clinical application. Future well-designed longitudinal studies using standardized diagnostic criteria, saliva collection procedures, and analytical methodologies are necessary to clarify the translational and clinical value of oxidative stress biomarkers in temporomandibular disorders. These findings support the potential role of salivary oxidative stress biomarkers as non-invasive tools for future personalized assessment and risk stratification in patients with temporomandibular disorder.

## Figures and Tables

**Table 1:** Characteristics of included studies.

Study	Country	Design	Participants	TMD Type	Diagnostic Criteria	Biomarkers Evaluated	Main Findings
Lawaf et al., 2015 [8]	Iran	Case–control	28 TMD with pain, 28 TMD without pain, 28 controls	Painful and painless TMD	Clinical TMD criteria	Serum TAC, Salivary TAC	Plasma TAC is significantly lower in TMD patients; salivary TAC is not significantly different
Omidpanah et al., 2020 [7]	Iran	Case–control	30 TMD, 30 controls	Pain-related TMD	RDC/TMD	Salivary MDA, TAC, Catalase	Salivary MDA is significantly higher in TMD; TAC and catalase are not significant
Vrbanović et al., 2019 [10]	Croatia	Longitudinal prospective study	12 female TMD patients	MP and DD	DC/TMD	TAC, MDA, SOD, UA, Cortisol	Reduction in MDA and MDA/SOD ratio after splint therapy
Klepzig et al., 2024 [12]	Germany	Randomized delayed-start trial	25 TMD, 11 controls	Chronic painful TMD	RDC/TMD	Cortisol, Alpha-amylase	Pain reduction associated with decreased cortisol after Michigan splint therapy

**Table 2:** Salivary oxidative stress biomarkers in TMD patients.

Study	Biomarker	TMD Group	Control Group	*p*-Value	Main Interpretation
Lawaf et al., 2015 [8]	Plasma TAC	0.89 ± 0.11 mmol/L	0.18 mmol/L	<0.001	Reduced systemic antioxidant capacity in TMD
Lawaf et al., 2015 [8]	Salivary TAC	Higher than controls	Lower	0.117	No significant salivary TAC alteration
Omidpanah et al., 2020 [7]	Salivary MDA	Higher than controls	Lower	0.001	Increased lipid peroxidation in TMD
Omidpanah et al., 2020 [7]	Salivary TAC	Higher than controls	Lower	0.22	Non-significant antioxidant reduction
Omidpanah et al., 2020 [7]	Catalase	Reduced after therapy	-	0.49	Non-significant antioxidant reduction
Vrbanović et al., 2019 [10]	Afternoon MDA	Reduced after therapy	-	0.021	Improvement after splint therapy
Vrbanović et al., 2019 [10]	MDA SOD Ratio	Reduced after therapy	-	0.017	Reduced oxidative imbalance
Vrbanović et al., 2019 [10]	TAC	Reduced after therapy	-	<0.05	Oxidative adaptation during treatment
Klepzig et al., 2024 [12]	Morning cortisol	Reduced after therapy	-	Significant	Stress-related biomarker improvement

**Table 3:** Quantitative synthesis of salivary oxidative stress biomarkers in TMD.

Biomarker	Number of Studies	Meta-Analysis Feasibility
MDA	1	Quantitative synthesis (single study)
TAC	2	Exploratory meta-analysis
Catalase	1	Narrative synthesis
SOD	1	Narrative synthesis
Cortisol	2	Narrative synthesis
Alpha-amylase	1	Narrative synthesis

**Table 4:** Risk of bias assessment (Newcastle–Ottawa Scale).

Study	Selection (4)	Comparability (2)	Exposure/Outcome (3)	Total Score (/9)	Quality
Lawaf et al., 2015 [8]	3	1	2	6/9	Moderate
Omidpanah et al., 2020 [7]	3	1	2	6/9	Moderate
Vrbanović et al., 2019 [10]	3	1	2	6/9	Moderate

**Table 5:** Methodological heterogeneity among included studies.

Study	Stimulated	Collection Time	Assay Method	Diag/Crit	Main Limitations
Omidpanah et al. (2020) [7]	Unstimulated	Morning	Spectrophotometry	DC/TMD	Small sample size
Lawaf et al. (2015) [8]	Unstimulated	Morning	Spectrophotometry	Clinical diagnosis	Lack of standardization
Vrbanović et al. (2019) [10]	Unstimulated	Morning	Elisa	DC/TMD	Follow-up variability
Klepzig et al. (2024) [12]	Unstimulated	Morning	Elisa	DC/TMD	Limited subgroup analysis

## Data Availability

No new data were created or analyzed in this study. Data sharing is not applicable to this article.

## Supplementary Materials

The following supporting information can be downloaded at: https://www.mdpi.com/article/10.3390/jpm16070357/s1. Table S1: Complete Electronic Search Strategies Used for Literature Retrieval; Table S2: PRISMA 2020 Checklist.
