# Supplementary material for: Salivary Oxidative Stress Biomarkers in Temporomandibular Disorders: A Systematic Review and Meta-Analysis
Source: J Pers Med. 2026 Jun 30;16(7):357. doi: 10.3390/jpm16070357 (PMC13413010; doi:10.3390/jpm16070357)
Supplement: Supplementary file 1 [file jpm-16-00357-s001.zip › Supplementary Table S1.pdf]

Supplementary Table S1. Complete Electronic Search Strategies Used for Literature Retrieval

The electronic search was performed in March 2026 in PubMed/MEDLINE, Embase, Scopus, and Web of Science. Search strategies were developed using a combination of controlled vocabulary terms and free-text keywords related to temporomandibular disorders, saliva, oxidative stress, and salivary biomarkers.

PubMed/MEDLINE

Search date: March 2026

("temporomandibular disorders"[MeSH Terms] OR "temporomandibular disorders" OR TMD OR "temporomandibular joint disorders") AND ("saliva" OR "salivary") AND ("oxidative stress" OR "malondialdehyde" OR MDA OR "total antioxidant capacity" OR TAC OR catalase OR "oxidative biomarkers")

('temporomandibular disorder'/exp OR 'temporomandibular disorder' OR TMD) AND ('saliva'/exp OR saliva OR salivary) AND ('oxidative stress'/exp OR 'malondialdehyde' OR MDA OR 'total antioxidant capacity' OR TAC OR catalase) TS= ("temporomandibular disorders" OR TMD) AND (saliva OR salivary) AND ("oxidative stress" OR malondialdehyde OR MDA OR "total antioxidant capacity" OR TAC OR catalase))

("temporomandibular disorders" OR "TMD" OR "temporomandibular joint disorders") AND ("saliva" OR "salivary") AND ("oxidative stress" OR "malondialdehyde" OR "MDA" OR "total antioxidant capacity" OR "TAC" OR "catalase")

Eligibility Framework

The search strategy was designed according to the PICO framework:

Component	Description
Population	Patients diagnosed with temporomandibular disorders (TMD)
Exposure	Oxidative stress and salivary oxidative biomarkers
Comparison	Healthy individuals without TMD
Outcomes	Quantitative salivary biomarker levels (MDA, TAC, CAT and related biomarkers)

Additional Search Procedures

- Reference lists of included studies were manually screened for potentially relevant articles.
- Duplicate records were removed before screening.
- Study selection was performed independently by two reviewers.
- Disagreements were resolved through discussion and consensus.
